# The complete mitochondrial genome for Stanley’s slug snake *Pareas stanleyi* (Serpentes: Pareidae) by next-generation sequencing

**DOI:** 10.1080/23802359.2021.1920508

**Published:** 2021-10-23

**Authors:** Xian Yang, Yu-Qian Shen, Jun-Jie Zhong, Li Wei, Zhi-Hua Lin, Li Ma

**Affiliations:** Laboratory of Amphibian Diversity Investigation, College of Ecology, Lishui University, Lishui, Zhejiang, China

**Keywords:** *Pareas stanleyi*, mitochondrial DNA, phylogenetic tree, next-generation sequencing

## Abstract

The complete mitochondrial DNA (mtDNA) for *Pareas stanleyi* was determined in this study. The length of complete mtDNA was 18,932 bp, including 13 protein-coding genes (PCGs) (*COI-III*, *ND1-6*, *ND4L*, *ATP6*, *ATP8* and *CYTB*), 23 tRNA genes, 2 rRNA genes, a L-chain replication-initiating non-coding region (NCR) and 2 control regions. The overall base composition of the sequence is 24.76% of T, 29.20% of C, 30.87% of A, and 15.16% of G, with a total A + T content of 55.63%. The phylogenetic tree showed that *P. stanleyi* had a close relationship with the other two species (*P. boulengeri* and *P. formosensis*) from the genus *Pareas*.

*Pareas* is a genus of Asian snakes in the family Pareidae, which contains 20 species (Bhosale et al. 2020). *Pareas stanleyi* is an endemic species, which is distributed in Fujian, Guizhou, Hunan, and Sichuan Provinces of China (Zhao 2006). In GenBank, only two complete mitochondrial DNA (mtDNA) are available for *Pareas* species, which are *P. boulengeri* (Huang et al. 2020) and *P. formosensis* (Liu et al. 2021). Here, we determined the complete circular mtDNA sequence of *P. stanleyi* via whole genomic sequencing, and found that it had a close relationship with the other species from the genus *Pareas.*

We collected the specimen (species voucher: LSU2020MLFJDT01) of *P. stanleyi* in Mount Wuyi National Park, from Nanping, Fujian Province, China (N27.71681°, E117.65694°) in July 2020. Presently, the specimen was stored in the Laboratory of Amphibian Diversity Investigation (ADI) at Lishui University (contact person: Li Ma, E-mail: lmahz2011@163.com). Total genomic DNA was extracted from the muscle tissue of *P. stanleyi* using EasyPure Genomic DNA Kit (TransGen Biotech Co, Beijing, China). The whole genomic sequences was ultrasonic fragmented, then sequenced by Illumina NovaSeq 6000 platform (Novogene Bioinformatics Technology Co. Ltd., Tianjin, China) for paired-150 bp. Raw sequence data (15.57G) were deposited in NCBI’s Sequence Read Archive (SRA accession: SRR13500293). The NOVO Plasty 3.7 was used to *de novo* assembled the clean data without sequencing adapters (Dierckxsens et al. 2017).

The mtDNA of *P. stanleyi* (GenBank accession no. MW531673) is a closed-circular molecule of 18,932 bp in length, which including 13 PCGs (*COX1-3*, *ND1-6*, *ND4L*, *ATP6*, *ATP8*, and *CYTB*), 23 tRNA genes, 2 rRNA genes, a L-chain replication-initiating non-coding region, and 2 control regions. The composition and arrangement of *P. stanleyi* mtDNA were approximately the same as *P. boulengeri* (Huang et al. 2020) and *P. formosensis* (Liu et al. 2021). The overall base composition of the sequence is 24.76% of T, 29.20% of C, 30.87% of A, and 15.16% of G, with a total A + T content of 55.63%. There are two duplicate genes of *tRNA^Leu^* and *tRNA^Ser^* in *P. stanleyi* mtDNA. All genes were encoded on the heavy strand except *ND6* and eight tRNA genes (*tRNA^Pro^*, *tRNA^Gln^*, *tRNA^Ala^*, *tRNA^Asn^*, *tRNA^Cys^*, *tRNA^Tyr^*, *tRNA^Ser^*, and *tRNA^Glu^*), which were encoded on the light strand. Among the 13 PCGs, the *ND5* was the longest (1788 bp), while the *ATP8* was the shortest (162 bp). Codon usage analysis of *P. stanleyi* showed that four kinds of start codons (ATA, ATG, ATT, and GTG) and six kinds of stop codons (AGA, AGG, TAA, TAG, TA-, and T–) were used. The 23 tRNA genes varied in size from 56 to 73 bp. The 12S rRNA (920 bp) and 16S rRNA genes (1487 bp) are located between *tRNA^Phe^* and *tRNA^Val^* and between *tRNA^Val^* and *tRNA^Ile^*, respectively.

Phylogenetic analysis was inferred from available mtDNA of *P. stanleyi* and other 24 snakes based on 13 concatenated PCGs (11,382 bp) with *Boa constrictor* as the outgroup using Bayesian inference (BI) method in MrBayes v3.2.2. The optimal substitution model (GTR + I + G) was implemented by MrModelTest 2.3 (Nylander 2004). We analyzed four parallel runs of Markov Chain Monte Carlo (MCMC) for 1,000,000 generations, sampling every 1000 generations and discarded 1000 trees as burn-in. The phylogenetic tree showed that *P. stanleyi* was the sister species of *P. boulengeri* within the genus of *Pareas* (Figure 1). The phylogenetic analysis result was consistent with the previous research. The genetic distance (*p*-distance) between *P. stanleyi* and *P. formosensis*, and between *P. stanleyi* and *P. boulengeri* was 20.16% and 18.44%, respectively, based on the 13 PCGs using MEGA 5.05 (Free Software Foundation, Boston, MA). The first determined mitogenome sequence of *P. stanleyi* will provide fundamental data for further exploring mDNA evolution in snakes.

**Figure 1.**
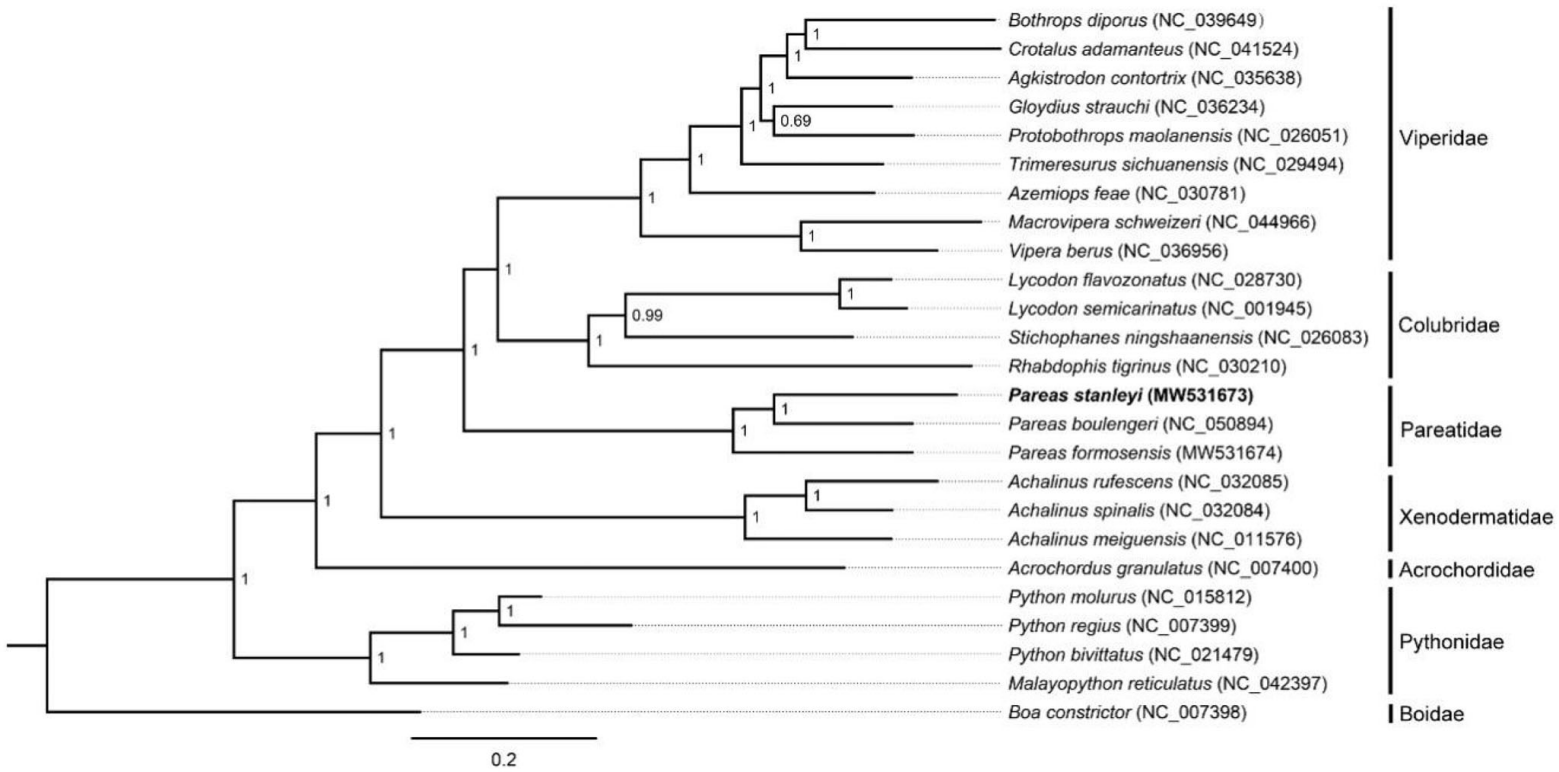
The phylogenetic tree (*P. stanleyi* and other 24 snakes) were analyzed with Bayesian inference (BI) method, based on 13 PCGs. The GenBank accession number are listed in the figure. Numbers at the nodes represent posterior probabilities.

## Data Availability

The mitogenome data supporting this study are openly available in GenBank at (https://www.ncbi.nlm.nih.gov/nuccore/MW531673). Reference number (Accession no. MW531673). BioSample and SRA accession numbers are https://www.ncbi.nlm.nih.gov/biosample/SAMN17393257 and https://www.ncbi.nlm.nih.gov/sra/SRR13500293, respectively.
